# Ageing potentiates diet-induced glucose intolerance, β-cell failure and tissue inflammation through TLR4

**DOI:** 10.1038/s41598-018-20909-w

**Published:** 2018-02-09

**Authors:** Wei He, Ting Yuan, Dolma Choezom, Hannah Hunkler, Karthika Annamalai, Blaz Lupse, Kathrin Maedler

**Affiliations:** Centre for Biomolecular Interactions, University of Bremen, Bremen, Germany

## Abstract

Ageing and obesity are two major risk factors for the development of type 2 diabetes (T2D). A chronic, low-grade, sterile inflammation contributes to insulin resistance and β-cell failure. Toll-like receptor-4 (TLR4) is a major pro-inflammatory pathway; its ligands as well as downstream signals are increased systemically in patients with T2D and at-risk individuals. In the present study we investigated the combined effects of high fat/high sucrose diet (HFD) feeding, ageing and TLR4-deficiency on tissue inflammation, insulin resistance and β-cell failure. In young mice, a short-term HFD resulted in a mildly impaired glucose tolerance and reduced insulin secretion, together with a β-cell mass compensation. In older mice, HFD further deteriorated insulin secretion and induced a significantly impaired glucose tolerance and augmented tissue inflammation in adipose, liver and pancreatic islets, all of which was attenuated by TLR4 deficiency. Our results show that ageing exacerbates HFD-induced impairment of glucose homeostasis and pancreatic β-cell function and survival, and deteriorates HFD-induced induction of mRNA expression of inflammatory cytokines and pro-inflammatory macrophage markers. TLR4-deficiency protects against these combined deleterious effects of a high fat diet and ageing through a reduced expression of inflammatory products in both insulin sensitive tissues and pancreatic islets.

## Introduction

Type 2 Diabetes mellitus (T2D) is a chronic metabolic disorder characterized by insulin resistance, a progressive decline in pancreatic β-cell function and mass and subsequent hyperglycaemia; all of which is strongly associated with obesity. A chronic, low-grade, “sterile” inflammation is present in obesity, and pro-inflammatory mediators including cytokines and ROS/RNS cause insulin resistance in peripheral insulin sensitive tissues and lead to dysfunction and apoptosis of insulin-producing β-cells in pancreatic islets^1,2^.

The innate immunity and especially tissue macrophages contribute to such obesity-associated inflammation^2,3^. Pro-inflammatory macrophages (termed as M1 or classically activated macrophages) infiltrate insulin responsive tissues; the adipose tissue, liver as well as pancreatic islets, and outnumber homeostasis-maintaining and anti-inflammatory tissue-resident macrophages (termed as M2 or alternatively activated macrophages)^4–6^. This, over time, leads to a chronic low-grade tissue inflammation, subsequent insulin resistance and loss in compensatory adaptation of the pancreatic β-cells with progression to hyperglycemia and diabetes^2,3^.

Clinical as well as preclinical experimental studies show that Toll-like receptor 4 (TLR4) expression and activation is directly associated with obesity-induced tissue inflammation; abrogation of TLR4 is able to reverse insulin resistance and pancreatic β-cell dysfunction in experimental models^7–14^. Hyperlipidemia alone or in concert with hyperglycemia, termed as “lipoglucotoxicity” can induce a pro-inflammatory state, shown in fat, where elevated free fatty acids lead to impaired insulin sensitivity. In pancreatic islets, prolonged lipoglucotoxicity initiates a vicious cycle in β-cell destruction^2,3^. Both of them are shown to be mediated by TLR4 signaling^4,6^.

TLR4 is a member of the TLR family of pattern recognition receptors, and its signaling is one of the major pro-inflammatory pathways. Two tightly connected pathways in obesity activate TLR4 through specific ligands and result in the exacerbation of inflammation; elevated free fatty acids (FFA) as well as lipopolysaccharide (LPS)-linked to changes in gut microbiota. Three major ligands of TLR4; LPS, CXCL10 and FFA are systemically increased in patients with T2D as well as in at-risk individuals^15–18^. While LPS is the known classical ligand of TLR4, FFA stimulates TLR4 signalling^19–21^, but rather than directly bound to TLR4, it acts through the hepatokine fetuin-A^22,23^, which is also increased in obesity^24^ and independently associated with T2D^25^. Plasma LPS levels are increased in rodent models of obesity as well as in obese individuals, this metabolic endotoxemia could be due to increased intestinal permeability and enhanced LPS absorption by HFD^26,27^. Other described TLR4 ligands increased in T2D patients include HMGB1, hyaluronan, Hsp60/70 as well as S100A8^16,28^.

Ageing is a major risk factor for the development of T2D, and is paralleled with developing glucose dys-homeostasis in both human and animal studies^29,30^. During ageing, a low-grade pro-inflammatory state has been observed with an elevation in pro-inflammatory cytokines, macrophages and superoxide products in fat, liver and pancreas, as well as in the circulation^30–35^. This may again be related to TLR4, as TLR4 mutant mice live longer, have stronger bones and muscles throughout their life^36^. In addition to diabetes, several other ageing-related diseases are mediated by increased inflammation through the pathological activation of TLR4, such as cardiovascular diseases, atherosclerosis, Alzheimer’s disease, arthritis and therefore, the term “inflamm-ageing” has been created to address such disease state with increased inflammation at an older age^37^.

Given this intersection of ageing, inflammation, TLR4 and diabetes, we hypothesized that ageing may have a potentiating effect upon obesity-induced tissue inflammation through TLR4, which would lead to an acceleration of the diabetes phenotype. Such possibility was addressed in the present study by short-term 8-week high fat/high sucrose diet-feeding of WT and TLR-4 knockout mice. We found that ageing could enhance diet-induced inflammatory cytokines in fat, liver and pancreatic islets, and aggravate impairment of glucose homeostasis and pancreatic β-cell dysfunction, which was prevented by TLR4-deficiency.

## Results

### **Ageing further impairs high fat diet induced glucose intolerance and insulin resistance in old WT but not in TLR4**^**−/−**^**mice**

**Figure 1:**
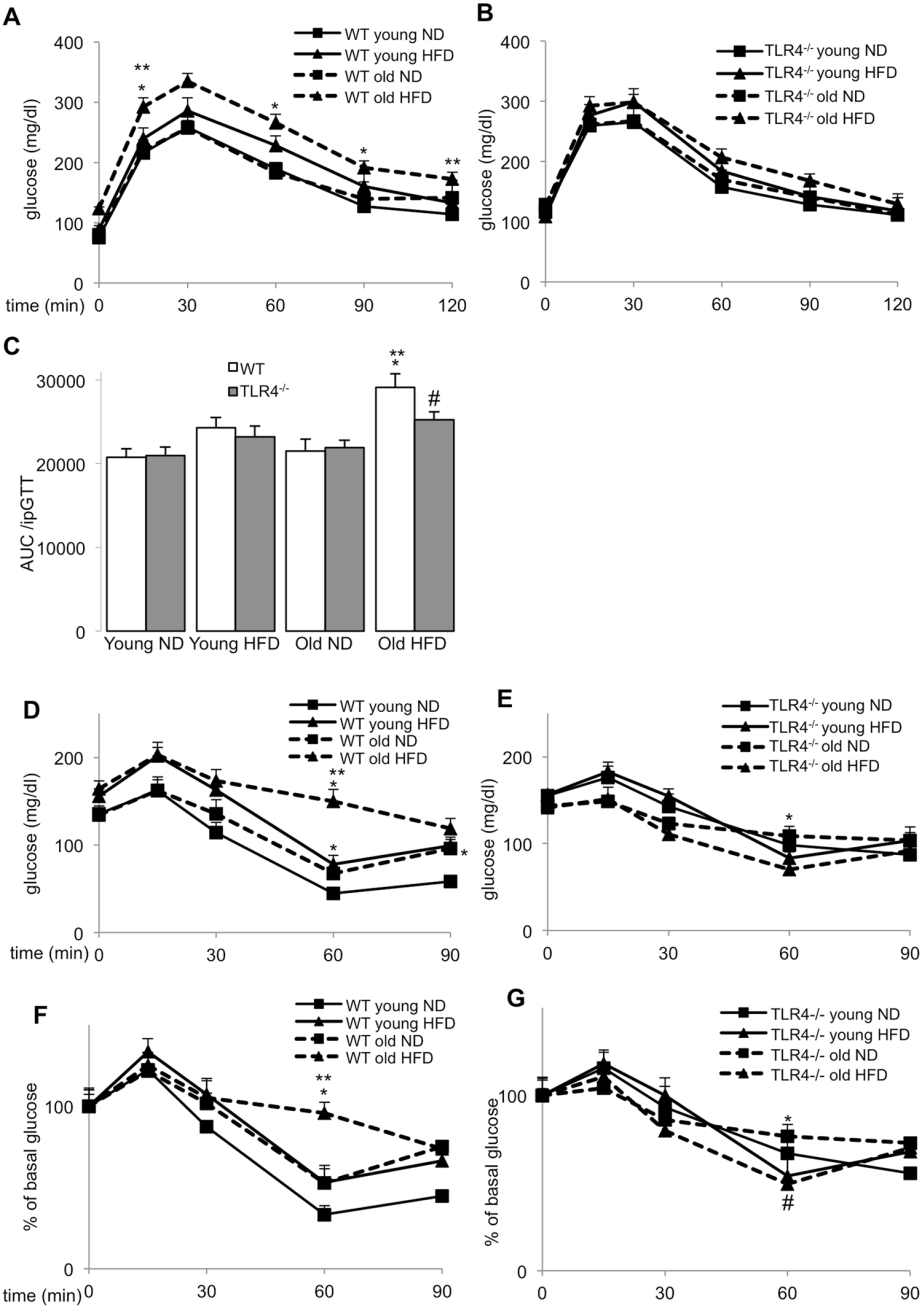
Ageing further impairs high fat diet induced glucose intolerance and insulin resistance in old WT but not in *Tlr4*^**−/−**^ mice **(A–G)** Young (6 weeks) and old (12 months) WT and *Tlr4*^−/−^ mice were fed a normal (ND) or high fat/high sucrose diet (“Surwit”; HFD) for 8 weeks. **(A–C)** Intraperitoneal glucose tolerance test (ipGTT) with 1 g/kg body weight glucose of WT **(A)** and *Tlr4*^−/−^ mice **(B)** and area-under-the-curve analysis by definite integrals of the same ipGTT results from the ND and HFD fed WT and *Tlr4*^**−/−**^ mice after 8 weeks of diet **(C). (D–G)** Intraperitoneal insulin tolerance test (ipITT) of WT **(D,F)** and *Tlr4*^−/−^ mice **(E,G)** with 0.75 IU/kg body weight insulin. Glucose levels were normalized to 100% before glucose injection **(F,G)**. Data are means ±SE. *p < 0.05 ND vs. HFD; **p < 0.05 young vs. old mice; ^#^p < 0.05 WT **(F)** vs. *Tlr4*^−/−^ **(G)** mice. Due to the congested figure, we split the data from WT and *Tlr4*^−/−^ mice used in the same experiments into two separate graphs (A/B, D/E, F/G). N = 12–15 mice per group; three independent experiments were performed.

To evaluate whether ageing potentiates hyperglycemia in obesity, we fed WT C57BL/6 J mice a normal (ND) or a high fat/high sucrose diet (HFD, “Surwit”). After 8 weeks of HFD feeding, WT mice at an age of 14 weeks, when they are usually investigated, developed obesity and a slightly impaired glucose tolerance, which were severely potentiated in HFD fed older mice of 14 months (Fig. 1A,C). In contrast, glucose tolerance was almost uncompromised in *Tlr4*^**−/−**^ mice of both ages (Fig. 1B,C).

These data are in line with the impaired insulin tolerance in HFD fed aged mice. While young mice developed only slightly impaired insulin tolerance under the HFD feeding, compared to ND, insulin resistance worsened in the aged mice (Fig. 1D,F). In *Tlr4*^**−/−**^ mice, insulin tolerance was unchanged -neither HFD nor ageing impaired insulin sensitivity during the 8-week feeding period (Fig. 1E,G). Body weight gain was significantly increased by the HFD, which was similar in both young and old WT and *Tlr4*^**−/−**^ mice (Suppl. Fig. 1A,B). Also food intake was not affected by age or by genotype (Suppl. Fig. 1C,D).

### **HFD led to β-cell failure in aged mice, whereas TLR4-depletion could restore β-cell function and survival**

**Figure 2:**
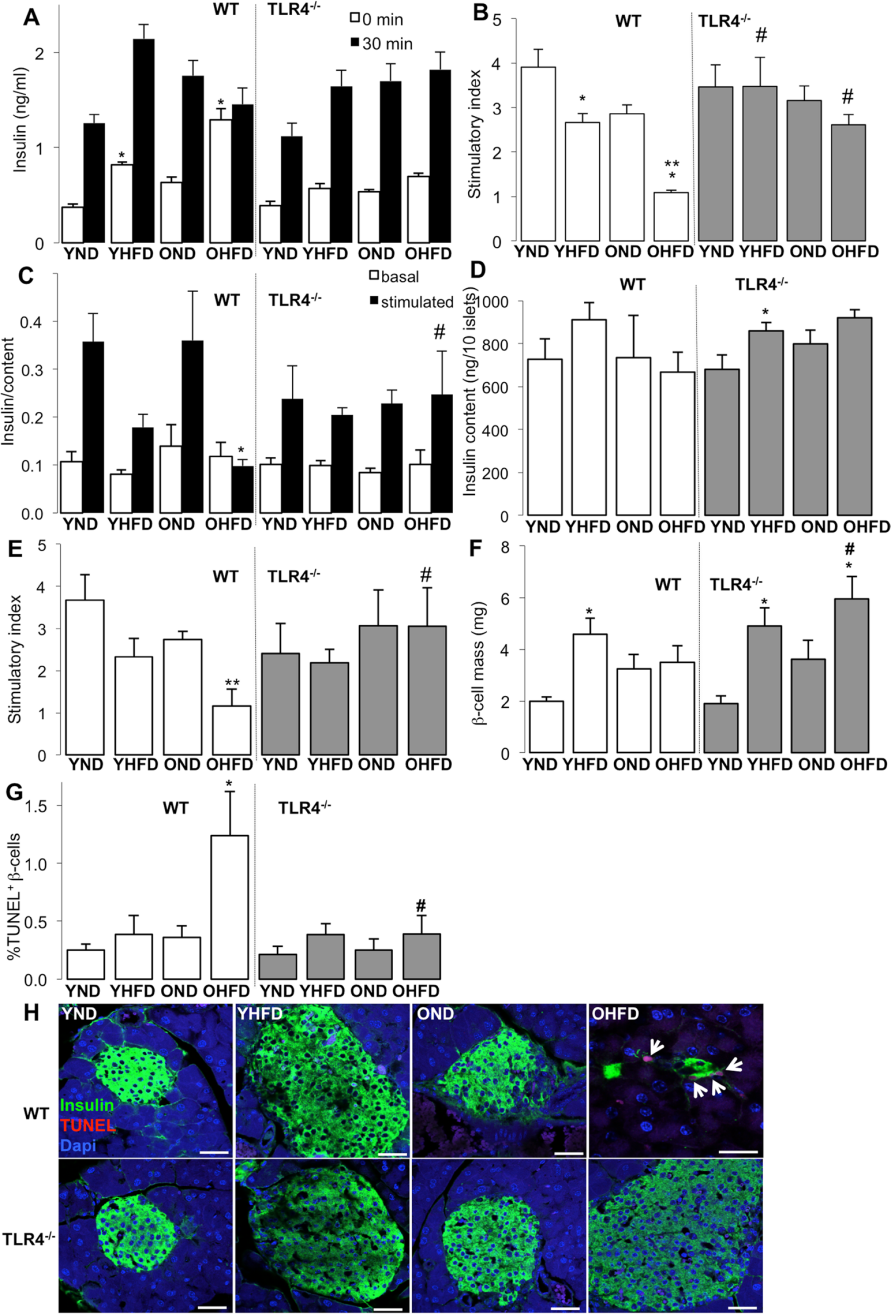
HFD led to β-cell failure in aged mice, whereas TLR4-depletion could restore β-cell function and survival. **(A–H)** Young (6 weeks) and old (12 months) WT and *Tlr4*^−/−^ mice were fed a normal (ND) or high fat/high sucrose diet (“Surwit”; HFD) for 8 weeks. **(A,B)** Insulin secretion during an ipGTT with 2 g/kg body weight glucose in week 8 measured before (0 min) and 30 min after glucose injection **(A)** and calculated as stimulatory index **(B)**. **(C–E)** Mice were sacrificed at week 8 and islets isolated from all 8 treatment groups, cultured overnight and subjected to an *in vitro* GSIS assay. **(C)** Insulin secretion during 1h-incubation with 2.8 mM (basal) and 16.7 mM glucose (stimulated), normalized to **(D)** insulin content. **(E)** The insulin stimulatory index denotes the ratio of secreted/basal insulin during 1h-incubation with 16.7 mM and 2.8 mM glucose, respectively. **(F)** β-cell mass analysed from 10 sections/mouse spanning the whole pancreas. **(G,H)** Results and representative microscopic images from triple staining for TUNEL, insulin and DAPI expressed as percentage of TUNEL-positive β-cells ±SE. The mean number of β-cells scored was 10,252 for each treatment condition. **(H)** Arrows point to four TUNEL^+^ β-cells with remaining insulin from an old HFD fed WT mouse. Scale bar, 50 μm. *p < 0.05 ND vs. HFD; **p < 0.05 young vs. old mice; ^#^p < 0.05 WT vs. *Tlr4*^−/−^ mice. N = 5–9 mice per group; three independent experiments were performed.

Since HFD and ageing led to an impairment of both glucose and insulin tolerance, we tested whether this may also be a result of impaired β-cell function and survival. Fasted mice were injected with 2 g/kg glucose, and insulin secretion was measured before (0 min) and 30 min after glucose injection. In parallel to the HFD induced hyperglycemia (Fig. 1A), WT HFD fed young as well as old mice (but not *Tlr4*^**−/−**^ mice) were hyperinsulinemic at the basal state (Fig. 2A), compared to ND mice.

The glucose stimulatory insulin secretion index was reduced in the young HFD mice and was fully abolished in the old HFD group, while it was fully maintained in *Tlr4*^**−/−**^ mice (Fig. 2B). Similar data were obtained from an *in vitro* GSIS assay, in which islets from all 8 groups were isolated and plated on extracellular matrix coated dishes for one day of recovery. While basal insulin secretion was unaffected in all groups, glucose stimulatory index was reduced by the HFD in islets from young mice and completely deprived in islets from old HFD fed mice (Fig. 2C,E). Again, *Tlr4*^**−/−**^ islets showed no impairment during the *in vitro* GSIS, and rescued high glucose induced insulin secretion in old mice fed a HFD compared to the wildtype littermates (Fig. 2C,E). Insulin content measured after the *in vitro* GSIS showed no significant changes among the 8 groups; except a significant increase in insulin content in the young *Tlr4*^**−/−**^ mice in adaptation to the HFD (Fig. 2D).

Previously, we reported a compensatory increase in β-cell mass in mice during the first 8 weeks of HFD feeding^38^. This was again confirmed in this study; HFD feeding induced a compensatory increase in β-cell mass in young mice, while old mice were unable to increase β-cell mass in response to the HFD (Fig. 2F). In contrast, both young and old *Tlr4*^**−/−**^ mice showed β-cell mass compensation (Fig. 2F). In line with the reduced β-cell mass, the number of apoptotic β-cells was increased in the old HFD fed mice (Fig. 2G), while apoptosis was significantly reduced in the old *Tlr4*^**−/−**^ mice fed a HFD, compared to the WT mice of the same group (Fig. 2G,H).

### **HFD-induced inflammatory cytokine expression was enhanced in old mice and attenuated by TLR4-deficiency**

**Figure 3:**
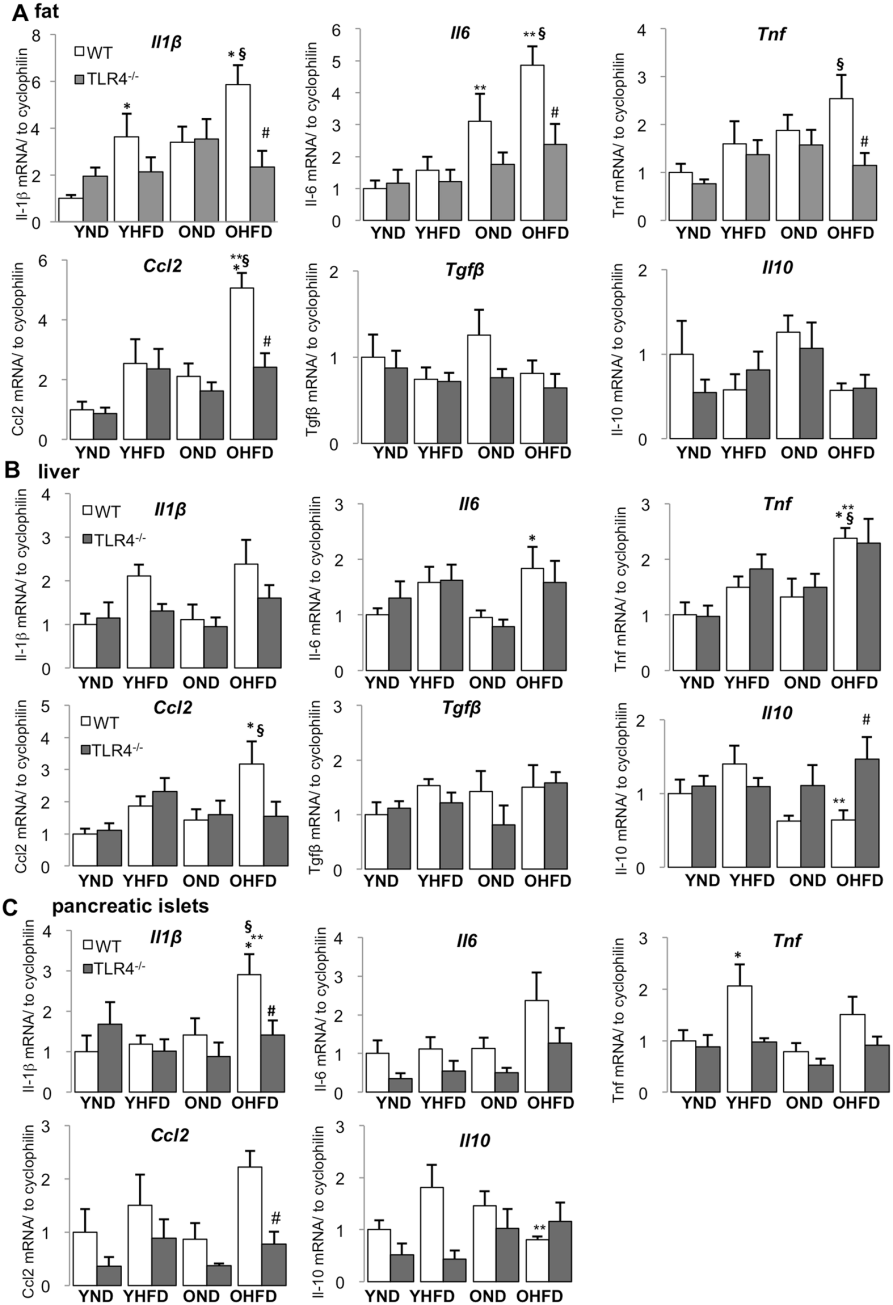
HFD-induced inflammatory cytokine expression was enhanced in old mice and attenuated by TLR4-deficiency. **(A–C)** Young (6 weeks) and old (12 months) WT and *Tlr4*^−/−^ mice were fed a normal (ND) or high fat/high sucrose diet (“Surwit”; HFD) for 8 weeks. RT-PCR analysis for inflammatory and anti-inflammatory genes with RNA extracted from fat **(A)**, liver **(B)** and pancreatic islets **(C)** of mice treated under the indicated conditions. Y, young mice, O, old mice, ND, normal chow diet, HFD, high fat/high sucrose diet, WT, wildtype mice, *Tlr4*^−/−^, TLR4-knockout mice. All results are normalized to control young-ND, which is arbitrarily set as 1. Data are presented as means ± SE from n = 5–8 mice. *p < 0.05 ND vs. HFD; **p < 0.05 young vs. old mice; ^#^p < 0.05 WT vs. *Tlr4*^−/−^ mice, ^§^p < 0.05 young ND vs. old HFD mice.

Multiple studies have revealed that HFD feeding and obesity can induce chronic low-grade tissue inflammation in fat, liver and pancreatic islets. In this study, we attempted to investigate, whether ageing augments cytokine expression induced by HFD feeding. In young mice, 8 weeks of HFD feeding induced *Il1b* expression in adipose tissue (Fig. 3A) and *Tnf* expression in pancreatic islets (Fig. 3C). In old mice, HFD feeding induced expression of *Il1b*, *Il6* and *Ccl2* in fat (Fig. 3A), *Il6*, *Tnf* and *Ccl2* in liver (Fig. 3B), *Il1b* in islets (Fig. 3C). While *Il1b* expression was already induced by HFD in fat of young mice, it was only induced in islets of older mice. Cytokine expression was also further accelerated in older mice on the HFD, especially in fat (e.g. *Il6* and *Ccl2*), but also in the liver, which expressed more *Tnf*, compared to HFD fed young mice. In general in all investigated tissues, the expression of multiple inflammatory cytokines was elevated by a combination of HFD and ageing when compared with young mice fed the control normal chow diet (Fig. 3A–C). Ageing itself induced a pro-inflammatory phenotype under the ND; seen by the significantly increased *Il6* expression in adipose tissue, but this was not observed in liver and islets (Fig. 3A–C).

Analysis of the anti-inflammatory cytokines *Il10*, *Tgfb* and *Il4* revealed that ageing together with HFD reduced *Il10* expression in liver and islets (Fig. 3A–C, Suppl. Fig. 2), while *Tgfb* levels remained unchanged under all conditions in fat and liver (Fig. 3A,B) and *Il4* was only detectable in liver but was unchanged by HFD and ageing (though TLR4 knockout showed a profound *Il4* induction in old HFD fed mice compared to their WT counterparts (Suppl. Fig. 2)).

Next, we checked if TLR4-deficiency affected such ageing/HFD induced cytokine expression and thus may explain the protective effect of TLR4 inhibition on glycemia, β-cell function and survival. Overall, TLR4-depletion had a tendency to reduce ageing/HFD induced pro-inflammatory cytokine expression (Fig. 3A–C). One exception is *Il6* and *Tnf* expression in liver, where was no change between *Tlr4*^**−/−**^ mice and WT mice, neither at ageing nor at HFD condition or both (Fig. 3B). Similarly, *Il10* expression was unchanged in fat and islets by TLR4-depletion, while it was increased together with *Il4* by TLR4-deficiency in the liver of old HFD mice (Fig. 3A,B and Suppl. Fig. 2).

These results indicate a very complex regulation of different cytokines in the insulin responsive and insulin-producing tissues, and that TLR4 is an important but unlikely the only pathway to initiate such cytokine expression pattern.

### **Combined HFD-feeding and ageing shifted tissue macrophage polarization to a more pro-inflammatory phenotype**

**Figure 4:**
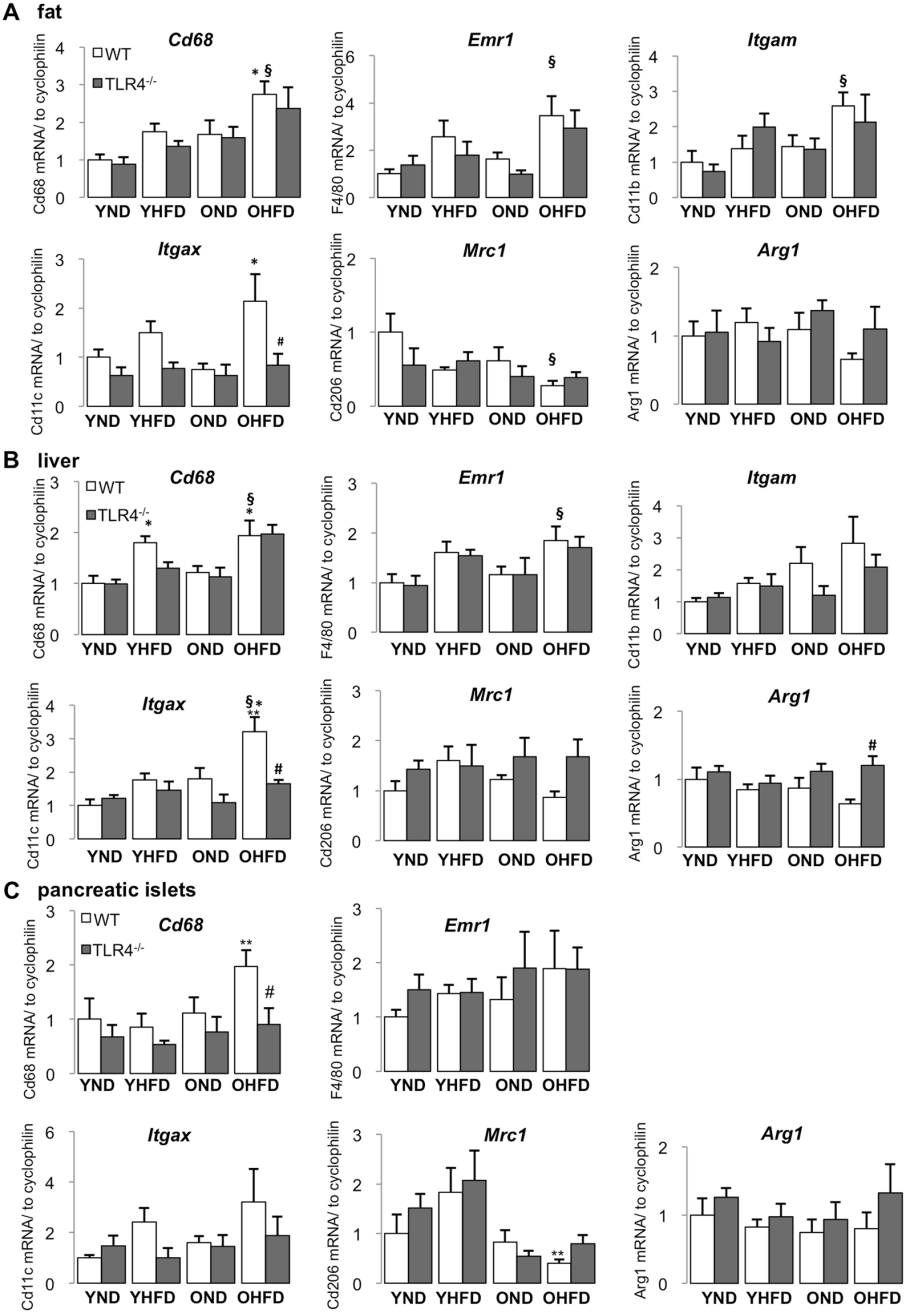
Combined HFD-feeding and ageing shifted tissue macrophage polarization to a more pro-inflammatory phenotype. **(A–C)** Young (6 weeks) and old (12 months) mice WT and *Tlr4*^−/−^ mice were fed a normal (ND) or high fat/high sucrose diet (“Surwit”; HFD) for 8 weeks. RT-PCR analysis for general macrophage and macrophage polarization markers with RNA extracted from fat **(A)**, liver **(B)** and pancreatic islets **(C)** of mice treated under the indicated conditions. Y, young mice, O, old mice, ND, normal chow diet, HFD, high fat/high sucrose diet, WT, wildtype mice, *Tlr4*^−/−^, TLR4-knockout mice. All results are normalized to control young-ND, which is arbitrarily set as 1. Data are presented as means ± SE from n = 5–8 mice. *p < 0.05 ND vs. HFD; **p < 0.05 young vs. old mice; ^#^p < 0.05 WT vs. *Tlr4*^−/−^ mice, ^§^p < 0.05 young ND vs. old HFD mice.

Since accumulation and classical activation of macrophages contributes to a pro-inflammatory phenotype observed in insulin sensitive and insulin-producing tissues under HFD feeding, we then analyzed macrophage markers in aged mice fed either ND or HFD. As general macrophage markers, we measured *Cd68*, *Emr1* (F4/80) and the pan-myeloid marker *Cd11b* (*Itgam*). We measured *Cd11c* (*Itgax*) as M1 macrophage marker^12,33,39,40^ and *Cd206* (mannose receptor, *Mrc1*) and *Arg1* (arginase 1) as M2 macrophage markers^9,12,40–42^. Comparison of young and old mice fed a ND revealed that ageing alone did not significantly change macrophage accumulation and polarization (Fig. 4A–C). Paralleled with the induction of inflammatory cytokines, the expression of general macrophage markers significantly increased by the combination of HFD and ageing in all tested tissues (Fig. 4A–C). In contrast, M2 markers showed the opposite; reduced *Mrc1* and *Arg1* expression, coinciding with the anti-inflammatory cytokine *Il10* (Fig. 3A–C), though detailed analysis revealed differences among tissues (Fig. 4A–C). Unlike in aged mice, the general macrophage markers were not uniformly induced by the 8-week HFD in young mice, only *Cd68* was increased in the liver (Fig. 4A–C).

Again, while an 8-week HFD regime triggers a minimal inflammatory profile in insulin responsive tissues and insulin-producing islets, ageing could potentiate HFD’s effect and augment tissue inflammation, macrophage accumulation and inflammatory activation.

Unlike the reduction in pro-inflammatory cytokine expression, TLR4 deficiency didn’t influence gene expression of macrophage accumulation and polarization in a universal manner. HFD induced *Itgax* expression was completely blocked in the liver and fat (Fig. 4A–C), and anti-inflammatory macrophage marker *Arg1* expression in liver of HFD fed old mice was enhanced by TLR4-deficiency (Fig. 4A–C), which is in line with the increased *Il4* and *Il10* expression in the same setting (Fig. 3B and Suppl. Fig. 2). The results above demonstrate that TLR4-deficiency could attenuate inflammatory macrophages in fat and liver, in addition to restoring M2-like macrophage polarization in the liver of old mice, both of which could contribute to prevent HFD-induced inflammation in old mice.

## Experimental Procedures

### Animals

C57BL/6 J (wild type; WT) and C57BL/10ScCr (TLR4 knockout; *Tlr4*^**−/−**^)^43^ male mice comprising of young (6 weeks) and old (12 months) mice were obtained from Jackson Laboratories (Bar Harbor, ME) and separated into 4 groups. Half of the groups were fed a normal diet (ND) (Harlan Teklad Rodent Diet 8604 containing 12.2, 57.6, and 30.2% calories from fat, carbohydrate, and protein, respectively; Harlan Teklad, Madison, WI) or a high fat/high sucrose diet (HFD) (“Surwit,” containing 58, 26, and 16% calories from fat, carbohydrate, and protein, respectively^44^; Research Diets, Inc., New Brunswick, NJ) for 8 weeks. Body weight and food intake were measured weakly during the study. All mice were housed in a temperature-controlled room with a 12 hours light, 12 hours dark cycle, and were allowed free access to food and water according to the protocol approved by the “Bremen Senate of Health” (the Institutional Animal Care and Use Committee) in agreement with the §8 of the German animal protection law. All methods were carried out in accordance with the guidelines and regulations of the Institutional Animal Care and Use Committee.

### Metabolic tests

For intraperitoneal glucose tolerance tests (ipGTT), mice were fasted 12 h overnight and injected i.p. with glucose (40%; B. Braun, Melsungen, Germany) at a dose of 1 g/kg body weight. Blood samples were obtained from the tail vein at time points 0, 15, 30, 60, 90 and 120 min for glucose measurements using a glucometer. Insulin secretion was measured before (0 min) and after (30 min) i.p. injection of glucose (2 g/kg body weight) with plasma taken from retro-orbital blood puncture using ultrasensitive mouse Elisa kit (ALPCO Diagnostics, Salem, NH) as described before^45^. For intraperitoneal insulin tolerance tests (ipITT), mice were fasted for 4 h and intraperitoneally injected with 0.75 IU/kg body weight of recombinant human insulin (InsHuman Rapid, Aventis, Germany) and blood glucose was measured 0, 15, 30, 60 and 90 minutes post injection.

### Mouse tissue isolation

After 8 weeks of diet, mice were sacrificed and islets were isolated by pancreas perifusion with Liberase^TM^ (Roche, Mannheim, Germany) according to the manufacturer’s instructions and digested for 10 minutes at 37 °C as previously described^45^. Islets were purified by a density gradient of Histopaque (1:1; 1077 and 1119, Sigma-Aldrich, Steinheim, Germany) and subsequent hand-picking. High purity islets were cultured overnight in RPMI 1640 medium containing 11.1 mM glucose (Lonza, Basel, Switzerland), followed by pelleting islets and adding Trizol (PEQLAB GmbH, Erlangen, Germany) for RNA extraction. Liver and epididymal white adipose tissue (WAT) were cut into small pieces in *RNAlater* solution (Sigma-Aldrich, Steinheim, Germany) and then shaken at 4 °C overnight, followed by homogenization and addition of Trizol for RNA extraction.

### Immunohistochemical analysis

Mouse pancreata were isolated and fixed with 4% PFA for 8 h at 4 °C and then paraffin embedded and cut into 4 μm sections. The slides were deparaffinized and immunostaining was carried out after heat antigen-retrieval^45^. Sections were incubated with anti-insulin (DAKO, Glostrup, Denmark; A0546, 1:100) for 12 h at 4 °C, and secondary antibody (biotin anti-guinea pig, 1:100; Jackson ImmunoResearch, PA) for 1 h at RT and thereafter with Vectastain ABC solution for 1 h at RT and DAB (HRP substrate kit, brown; both from Vector Laboratories, Burlingame, CA, USA) for 10 min. For morphometric analysis, ten sections (spanning the width of the pancreas) per mouse were analyzed as described before^45^. Pancreatic tissue area and insulin-positive area were determined by computer-assisted measurements using a Nikon MEA53200 (Nikon GmbH, Dusseldorf, Germany) microscope and images were acquired using NIS-Elements software (Nikon GmbH, Dusseldorf, Germany). Mean percent β-cell fraction per pancreas was calculated as the ratio of insulin-positive and whole pancreatic tissue area. β-cell mass was obtained by multiplying the β cell fraction by the weight of the pancreas. β-cell apoptosis was analyzed by the TUNEL technique according to the manufacturer’s instructions (*In situ* Cell Death Detection Kit, TMR red; Roche/now distributed by Sigma-Aldrich, Steinheim, Germany) and double stained for insulin, followed by FITC anti-guinea pig secondary antibody (Jackson).

### Glucose-stimulated insulin secretion (GSIS) *in vitro*

For acute insulin release in response to glucose, primary mouse islets were washed and pre-incubated (30 min) in Krebs-Ringer bicarbonate buffer (KRB) containing 2.8 mM glucose and 0.5% BSA. KRB was then replaced by KRB 2.8 mM glucose for 1 h (basal), followed by an additional 1 h in KRB 16.7 mM glucose (stimulated). Thereafter, islets were washed with PBS and extracted with HCl (0.18 N) in 70% ethanol overnight at 4 °C. The acid-ethanol extracts were collected for determination of insulin content. Insulin was determined using mouse insulin ELISA (ALPCO Diagnostics, Salem, NH). Secreted insulin was normalized to total insulin content.

### RNA extraction and quantitative RT-PCR analysis

Total RNA was isolated from mouse islets, liver and WAT with a Trizol extraction system (TriFast, PEQLAB GmbH, Erlangen, Germany). cDNA synthesis and quantitative RT-PCR was performed as previously described^45^. The Applied Biosystems StepOne Real-Time PCR system (Applied Biosystems, CA) with TaqMan® Fast Universal PCR Master Mix for TaqMan assays (Applied Biosystems, CA) was used for analysis. Cyclophilin (PPIA) and β-Actin (ACTB) were used as internal housekeeping controls and the quantitative analysis was performed with the ΔΔCT method. The following TaqMan® Gene Expression Assays were used: *Ppia* (Mm03024003_g1), *Actb* (Mm00607939_s1), *Il1b* (Mm00434228), *Il6* (Mm00446190), *Tnf* (Mm00443258_m1), *Ccl2* (Mm00441242_m1), *Il4* (Mm00445259_m1), *Il10* (Mm00439614_m1), *Tgfb* (Mm01178820_m1), *Cd68* (Mm03047343_m1), *Emr1* (F4/80) (Mm00802529_m1), *Itgam* (CD11b) (Mm00434455_m1), *Itgax* (CD11c) (Mm00498698_m1), *Mrc1* (CD206) (Mm00485148_m1), *Arg1* (Mm00475988_m1).

### Statistical analysis

All values were expressed as means ± SE with the number of independent individual experiments (*in vitro*; biological replicates) or the number of mice (*in vivo*) presented in the figure legends. The different groups were compared by two-way ANOVA with Bonferroni post-tests. P value < 0.05 was considered statistically significant.

## Discussion

The present study identifies a deleterious potentiation of impaired glucose homeostasis, β-cell dysfunction and chronic tissue inflammation by the combination of obesity and ageing. Data from our HFD/ageing mouse model support the concept that obesity with ageing leads to further deterioration in blood glucose regulation, which is likely due to a reduced capacity to balance inflammatory genes at an older age.

While our previous studies investigated the effect of chronic HFD feeding on glucose homeostasis and stages of β-cell adaptation, compensation and failure^46^, in this study we exposed the mice with the HFD for a relatively short term of 8 weeks, which only slightly impaired glucose and insulin tolerance in young mice of around 14 weeks, an age when they are usually investigated, but this was apparently exacerbated in older mice of 14 months of age.

Results from the *in vivo* GSIS displayed that β-cell function was impaired by the HFD regardless of age, but the definite amount of secreted insulin was only reduced in aged mice, while young mice could compensate for the increased insulin demand at a mildly insulin resistant stage. This suggests that β-cells from young mice keep a sufficient plasticity to maintain its insulin secretion function. This is also reflected by the changes in β-cell mass and in line with previous data showing that the ability of β-cells to proliferate is lost during ageing^47,48^. Young but not old mice respond to a HFD with β-cell mass expansion to meet the increased insulin demand. Such differences in the β-cell expansion capacity are not only observed with ageing but also with duration of diet. While high fat diet feeding for up to 8 weeks results in β-cell mass expansion, such adaptive increase in β-cell mass is not observed any more after 12 weeks^38^. This also correlates with β-cell apoptosis, which is only seen after longer periods of HFD feeding^45^, while after 8 weeks, HFD feeding does not result in changes of β-cell survival in young mice, but induces β-cell apoptosis in old mice. Such effects are certainly also dependent on the diet composition, as an even higher carbohydrate content in the diet (35% of calorie intake) can already severely impair glucose homeostasis in young mice^49^. While the diabetogenic “Surwit diet” of 58, 16 and 26% calories from fat, protein and carbohydrate, respectively^44^, disrupts insulin secretion later in life, β-cells can compensate for this in young mice. Their survival is not significantly affected yet, and there is only minor deterioration in glucose and insulin tolerance. This is reminiscent of our earlier study in human islets *ex vivo*, which shows that β-cell survival *per se* is not impaired in older individuals, but in response to diabetogenic stimulation, such as HFD or hyperglycemia, apoptosis is accelerated^47^. In aged HFD mice, there is a significantly elevated basal insulin secretion, and subsequently insulin secretion cannot be further induced in response to glucose during *in vivo* GSIS, which was just recently confirmed^50^. Such elevated basal insulin level is mainly due to insulin resistance and elevated FFA levels during an obese insulin resistant stage. The improvement in the β-cell stimulatory index by TLR4 deficiency is mainly attributed to the normalization in basal secretion. Interestingly, despite no obvious impairment in insulin sensitivity by the HFD in TLR4-KO mice, there is a compensatory increase in β-cell mass and insulin content, which suggests that such adaptation may be independent of insulin sensitivity.

In an *in vitro* GSIS assay, in which the effect of insulin resistance can be ruled out, basal insulin levels were similar and glucose stimulated insulin secretion reduced in young HFD mice, and fully abolished in old HFD mice, suggesting the secretory function is also compromised, while TLR4 deficiency protected the islets from such functional depletion. Such obvious loss in the secretory function was also observed in islets from senescent (21–22-month old) Fisher rats, compared to young rats (4–5-month old)^51^. Also in 7–8-month old Wistar rats, insulin production as well as secretion is impaired^47^, which is attributed at least in part to the reduction in PDX1^47,52^, the factor for glucose mediated insulin production in mature β-cells. Other factors, which lead to an almost complete decline in β-cell regeneration in ageing are the increased expression of the cell cycle inhibitor P16^48^, which is initiated by decreased Bmi-1 binding to the Ink4a/Arf locus^53^ and by decreased Ezh2^54^; both increase P16, and thus disable β-cell proliferation. As cell cycle and senescence markers have been identified in islets during ageing, we specifically focused here on markers of the inflammatory response; not only in islets but also in insulin responsive tissues. Overall, our study indicates that a mild ageing itself doesn’t induce β-cell functional impairment and survival, whereas it can potentiate the adverse effects of a short-term HFD.

“Sterile” chronic, low-grade inflammation without any obvious infection is a common feature of ageing, and people over the age of 65 have increased serum levels of IL-6, TNF, and IL-18^55,56^. Similarly, in rodent models of ageing, IL-1β, IL-6, MCP-1 (CCL2), TNF and IL-12b increase in fat and liver^31,33,34^. With respect to the pancreas, oxidative stress increases in aged mouse pancreases *per se* at the age of 14–16 months^32^ and TNF expression is elevated in pancreatic acinar cells in female mice aged 18–19 months^35^. In the present study, however, a pro-inflammatory cytokine expression by ageing alone was only seen in fat, but not in liver and islets, though differences in age, species and strains exist among this and other studies. We show that ageing alone neither induced metabolic deterioration nor an overall activated inflammatory state in metabolically active tissues.

Along with inflammatory cytokines, lipotoxicity contributes to insulin resistance and β-cell dysfunction through oxidative stress^57^. Free fatty acids activate TLR4, which further downstream leads to ROS/RNS production^58^. This is likely another mechanism, besides the inhibition of inflammation, by which TLR4 depletion ameliorated glucose homeostasis, β-cell function and survival in aged HFD fed mice in this study, even though we have not addressed such possible mechanism.

To the question whether the number of macrophages increase in tissues during ageing, several studies reported macrophage accumulation in fat, liver and/or pancreas of aged mice or rats^30,31,34^, although this was not confirmed by others^32,33^. In our current study, based on gene expression of accepted markers, neither macrophage accumulation nor their polarization status was changed in older mice in any of the three tissues, which is in line with the unchanged tissue inflammation. Mutually contradictive results were obtained from various studies, where down-regulation in both M1 and M2 polarization markers^59^, increased M2 macrophages^60^, or an overall macrophage polarization towards the M1 type during aging were observed^33^. The problem is that different age groups were used in these studies, with young animals ranging from 1–6 months and old animals ranging from 12–24 months age, thereby no corresponding correlation between an older age and worsening of the inflammatory phenotype could be drawn.

HFD feeding in the young mice for a short period of 8 weeks did not induce a full cytokine response at the mRNA level. The first elevated cytokines in response to the HFD were *Il1b* in fat and *Tnf* in islets. Since we didn’t observe compromised glucose tolerance, the very low-grade inflammation in young mice is consistent with the consensus that inflammation precedes hyperglycemia. In contrast, insulin secretion in young mice, tested by GSIS *in vivo* as well as *in vitro*, was already affected at this stage, together with the β-cell mass compensation response. *Tnf* was the only cytokine, which was induced in pancreatic islets by the short HFD feeding in the young mice, and this could act as mediator of β-cell dysfunction. This is in line with a previous study; while TNF does not affect β-cell apoptosis, it blunts GSIS from sorted β-cells^61^. Such results point to the possibility of early detrimental effects on β-cell function mediated by TNF. Especially, TNF is known to trigger insulin resistance and was also highly elevated at an insulin resistant stage in liver and fat in the old HFD mice. Thus, the results of this study also support the strategy to target TNF for the treatment of insulin resistance and β-cell failure^62^.

Neither ageing nor short term HFD itself induced severe hyperglycemia or massive changes in the cytokine pattern. But the combination of both synergistically induced inflammation in all insulin responsive and insulin secreting tissues- fat, liver and pancreatic islets. Along with hyperglycemia, insulin resistance, fully abolished insulin secretion and β-cell apoptosis, tissues were more inflamed in old HFD mice than in young, including a more and stronger pro-inflammatory and reduced anti-inflammatory cytokine expression. One limitation of this study is, that we only assessed mRNA levels of inflammatory products, which allowed quantitative analysis of cytokines at a very low expression levels. Moreover, cytokines are unstable and degrade rapidly, and thus often lay under the assay detection range, which makes their assessment on a protein level in tissues difficult.

Consistent with cytokine profiles, we found that only the combination of ageing and HFD feeding could increase the overall macrophage accumulation, again in line with the finding that ageing could potentiate HFD-induced gene expression of inflammatory cytokines, of markers of pro-inflammatory macrophages, along with a reduction in anti-inflammatory macrophage markers in fat and islets, metabolic dysfunction and β-cell failure.

A scenario emerges, how the HFD-ageing duo affects glucose metabolism: young mice are responsive to HFD with mildly increased macrophages in metabolism active tissues. This contributes to a mild inflammatory cytokine production and, in turn, results in an impairment of β-cell function. However, β-cells are still resilient to maintain compensation and glucose homeostasis. When mice get older, the same short-term diet stress not only increases M1-like macrophages but also attenuates M2-like macrophage activation, which further imbalances the macrophage phenotype and brings deterioration in the inflammatory status with more pro- and less anti-inflammatory cytokines. This then may lead to insulin resistance and compromised β-cell function, and in combination with reduced β-cell proliferation and increased β-cell apoptosis during ageing, it will finally result in definite insulin deficiency and concomitant hyperglycemia.

Being a crucial pattern recognition receptor and key player in inflammation, TLR4 is involved in many aspects of the pathogenesis of T2D, at the level of both β-cells and insulin responsive tissues^7,8,10,13^. As we aimed to identify the contribution of TLR4 on whole body glucose metabolism, we used whole-body TLR4-KO mice. This strategy, however, restrained the identification of the primary tissues affected by TLR4 signals. The generation of mouse models with tissue specific TLR4 re-expression in adipocytes/hepatocytes/β-cells/macrophages, respectively, on a TLR4-KO C57BL/10ScCr background would allow characterization of tissue specific effects, as well as a proof of a TLR4 specific effect upon its re-expression.

Tissue specific TLR4 effects have been studied in the past and confirmed observation from global deletions, e.g. myeloid-specific (as well as global) TLR4-deficiency improves insulin sensitivity and inhibits obesity-induced tissue inflammation in HFD and lipid infusion models^7,11,22,63,64^. However, in TLR4-deficienct mice, both reduced^7,11,65^ and unchanged ATM accumulation has been reported^9,64^. Notably, Orr *et al*. found that TLR4-depletion promoted M2 polarization in fat^9^. Similarly, Jia *et al*. observed that myeloid-specific *Tlr4*^**−/−**^ had a trend to promote macrophage alternative activation in fat together with induced IL-10 production^12^.

Featuring the combinational effect of a mild HFD and ageing, our results in insulin-responsive and insulin-producing tissues from old HFD mice indicate an overall trend that TLR4-deficiency reduces mRNA expression of inflammatory cytokines and M1 macrophage markers, and additionally promotes alternative macrophage activation specifically in the liver. This *in vivo* study is also in line with previous *ex vivo* studies^13,66^; TLR4 activation in isolated islets induces cytokine expression, impairs glucose-stimulated insulin secretion and increases β-cell apoptosis. This is again supportive for the role of TLR4 activation in diabetes progression.

In summary, we found that ageing aggravated diet-induced impairment on glucose homeostasis, pancreatic β-cell function and survival and enhanced gene expression of inflammatory products in fat, liver and pancreatic islets in a HFD-fed mouse model. TLR4-deficiency exhibited protection against such deleterious effects through inhibiting pro-inflammatory cytokine expression and modulating tissue macrophage activation to a more anti-inflammatory phenotype. Ageing and obesity synergistically induce diabetes through TLR4, supporting the therapeutic potential of TLR4 inhibition to treat T2D.

## Electronic supplementary material

Supplementary Information
