# Supplementary material for: Ageing potentiates diet-induced glucose intolerance, β-cell failure and tissue inflammation through TLR4
Source: Sci Rep. 2018 Feb 9;8:2767. doi: 10.1038/s41598-018-20909-w (PMC5807311; doi:10.1038/s41598-018-20909-w)
Supplement: Supplementary file 1 — Supplementary Information [file 41598_2018_20909_MOESM1_ESM.docx]

**Ageing potentiates diet-induced glucose intolerance, β-cell failure and tissue inflammation through TLR4**

Wei He, Ting Yuan, Dolma Choezom, Hannah Hunkler, Karthika Annamalai, Blaz Lupse and Kathrin Maedler

Centre for Biomolecular Interactions, University of Bremen, Bremen, Germany

**
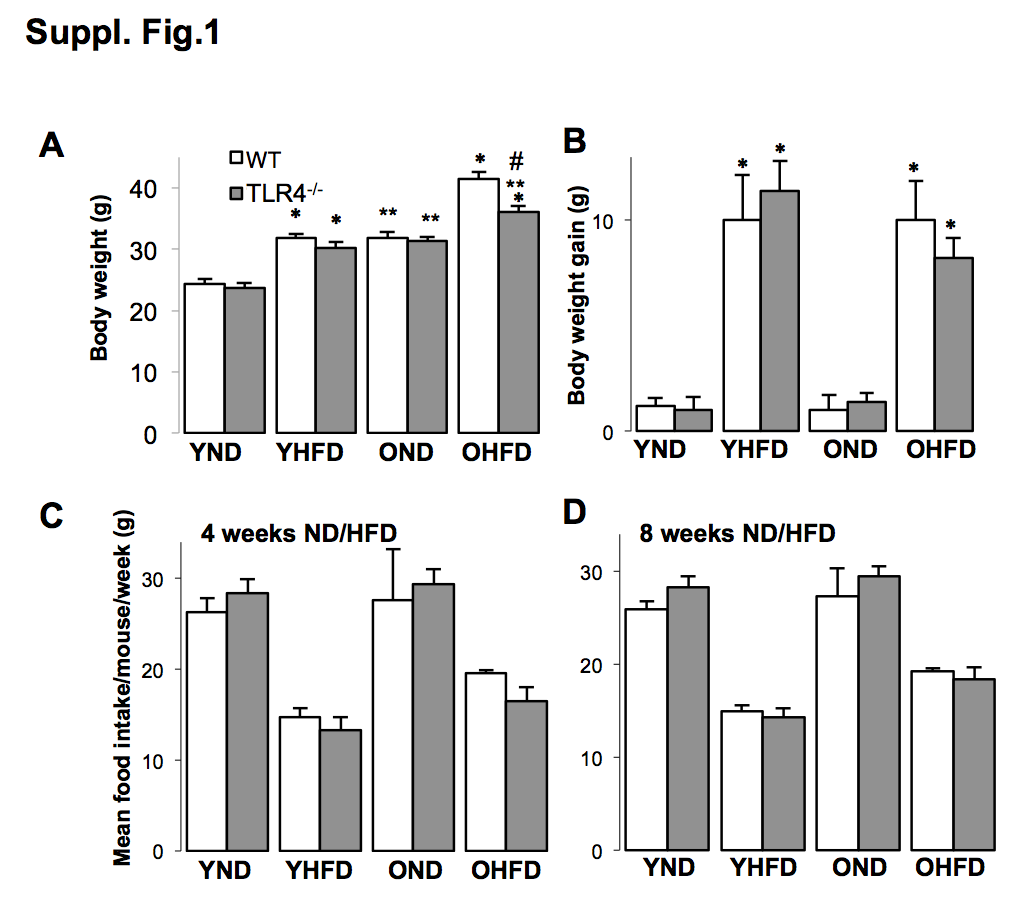
Suppl.Figure 1. HFD induced body weight gain and stable food intake in mice of both ages and genotypes.**

(A) Body weight, (B) body weight gain and (C,D) mean food intake/mouse/week after 8-week normal (ND) or high fat/ high sucrose diet (“Surwit”; HFD) feeding of young (6 weeks) and old (12 months) WT and *Tlr4^-/-^*mice. (C,D) Food intake was stable among the mice and unchanged with weeks of diet. Mice fed a HFD eat constantly a lesser amount of food. Data are shown as means ±SE. *p<0.05 ND vs. HFD; **p<0.05 young vs. old mice, #p<0.05 WT vs. *Tlr4^-/-^*mice. N=12-15 mice per group; data are representative of three independent experiments.

*
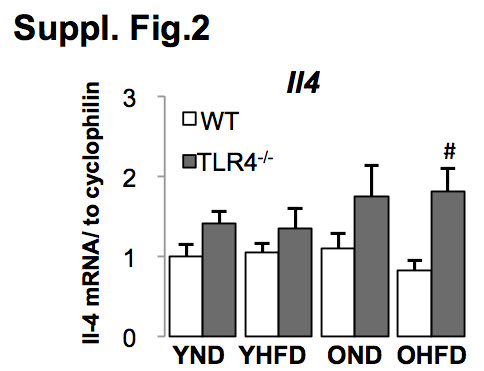
***Suppl.Figure 2. *Tlr4^-/-^* mice showed higher anti-inflammatory cytokine *Il4* expression in liver**

Young (6 weeks) and old (12 months) WT and *Tlr4^-/-^* mice were fed a normal (ND) or high fat/ high sucrose diet (“Surwit”; HFD) for 8 weeks. RT-PCR analysis of *Il4* from RNA extracted from liver. Y, young mice, O, old mice, ND, normal chow diet, HFD, high fat/ high sucrose diet, WT, wildtype mice, *Tlr4^-/-^*, TLR4-knockout mice. Results are normalized to the control young-ND condition, which is arbitrarily set as 1. Data are presented as means ±SE from n=5-8 mice. #p<0.05 WT vs. *Tlr4^-/-^* mice.
